# Supplementary material for: The Coordinated Interplay Between MMP13 and Pro-Migratory MMPs in Collective Cell Migration of Zebrafish Keratocytes
Source: Int J Mol Sci. 2025 Nov 19;26(22):11192. doi: 10.3390/ijms262211192 (PMC12652764; doi:10.3390/ijms262211192)
Supplement: Supplementary file 1 [file ijms-26-11192-s001.zip › Supplemental Figures 1 and 2.pptx]

## Slide 1
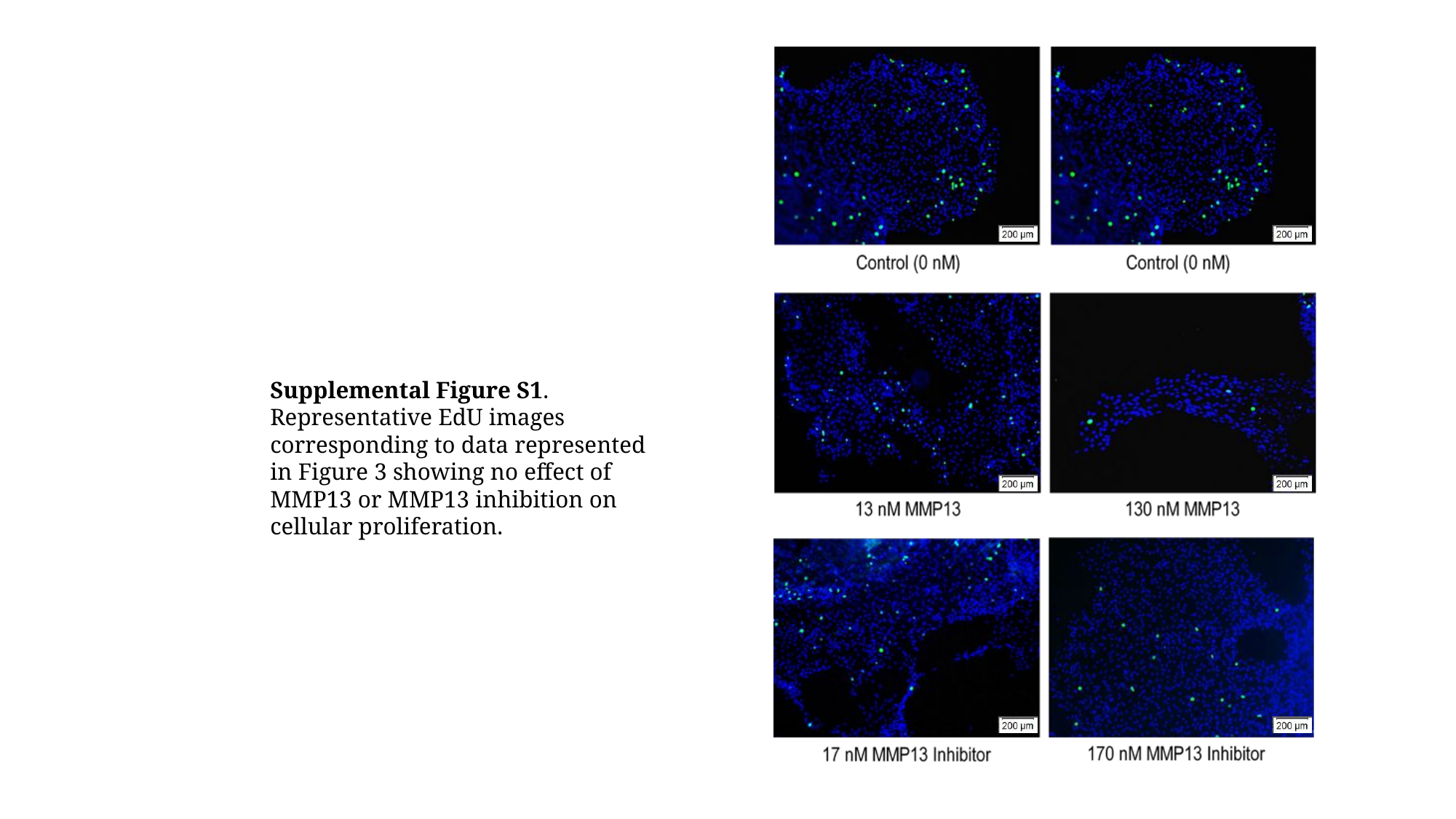

Supplemental Figure S1. Representative EdU images corresponding to data represented in Figure 3 showing no effect of MMP13 or MMP13 inhibition on cellular proliferation.

## Slide 2
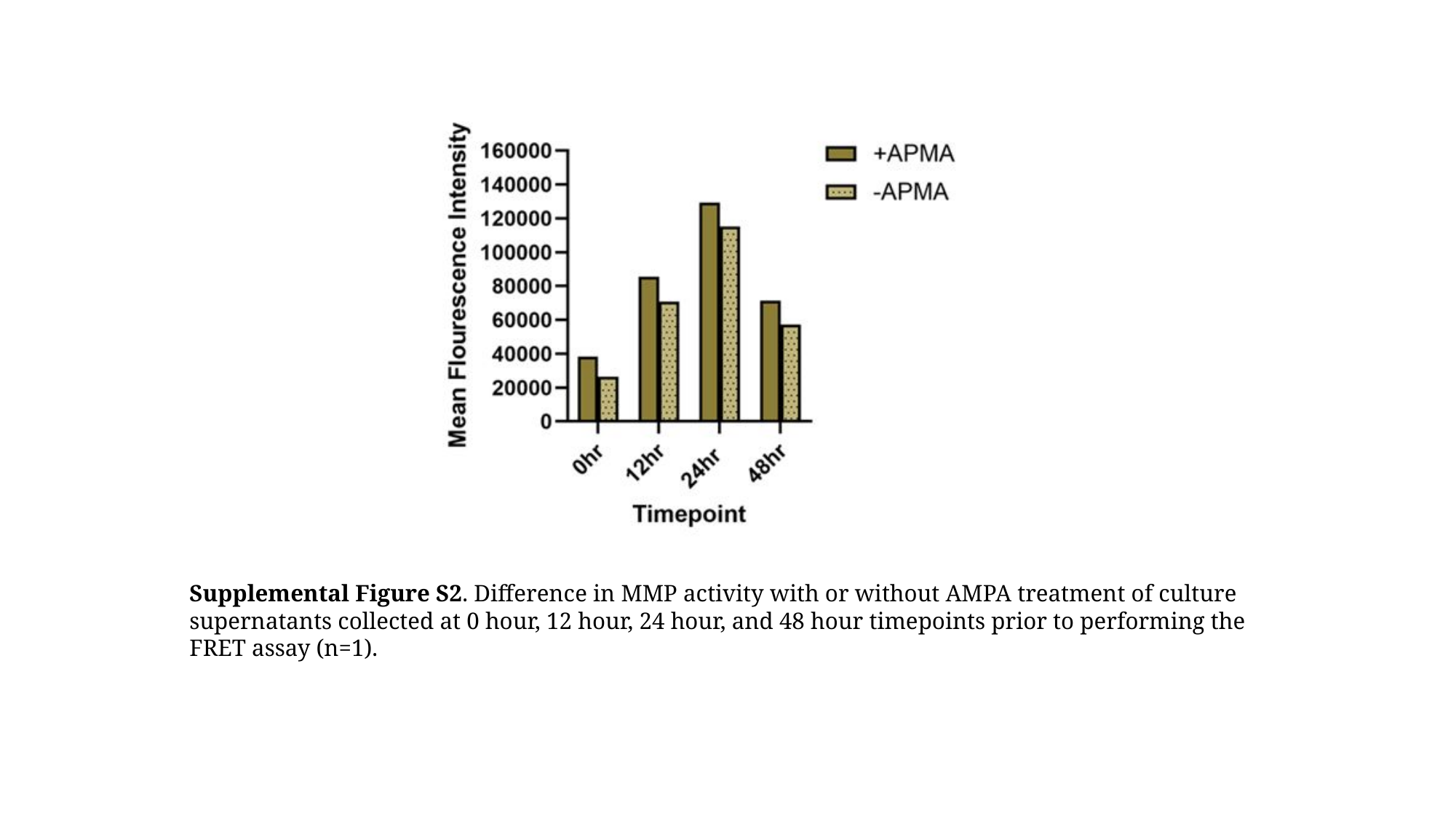

Supplemental Figure S2. Difference in MMP activity with or without AMPA treatment of culture supernatants collected at 0 hour, 12 hour, 24 hour, and 48 hour timepoints prior to performing the FRET assay (n=1).
